# Supplementary material for: Unveiling the Metal-Dependent Aggregation Properties of the C-terminal Region of Amyloidogenic Intrinsically Disordered Protein Isoforms DPF3b and DPF3a
Source: Int J Mol Sci. 2022 Dec 4;23(23):15291. doi: 10.3390/ijms232315291 (PMC9738585; doi:10.3390/ijms232315291)
Supplement: Supplementary file 1 [file ijms-23-15291-s001.zip › ijms-2043267-supplementary.pdf]

Supporting Information

–

Unveiling the metal-dependent aggregation properties of the C-terminal region of amyloidogenic intrinsically disordered protein isoforms DPF3b and DPF3a

Tanguy Leyder[1,†], Julien Mignon[1,2,3,†], Denis Mottet[4], and Catherine Michaux[1,2,3,*]

[1]*Laboratoire de Chimie Physique des Biomolécules, UCPTS, University of Namur, 61 rue de Bruxelles, 5000 Namur, Belgium*

[2]*Namur Institute of Structured Matter (NISM), University of Namur, Namur, Belgium*

[3]*Namur Research Institute for Life Sciences (NARILIS), University of Namur, Namur, Belgium*

[4]*GIGA-Molecular Biology of Diseases, University of Liège, Quartier Hôpital, Avenue de l'Hôpital 11, 4000 Liège, Belgium*

*Corresponding author: Catherine Michaux, Laboratoire de Chimie Physique des Biomolécules, UCPTS, Université de Namur, rue de Bruxelles 61, 5000 Namur, Belgique. Phone number: +32 81724557. E-mail address: catherine.michaux@unamur.be

[†]These authors contributed equally to this study

C-TERb:

```
                 200        210        220        230        240        250        260        270
                   C DICGKRYKNR PGLSYHYAHT HLASEEGDEA QDQETRSPPN HRNENHRPQK GPDGTVIPNN YCDFCLGGSN

       280        290        300        310        320        330        340        350        360
MNKKSGRPEE LVSCADCGRS GHPTCLQFTL NMTEAVKTYK WQCIECKSCI LCGTSENDDQ LLFCDDCDRG YHMYCLNPPV AEPPEGSWSC

       370
HLCWELLKEK ASAFGCQA
```

C-TERa:

```
                 200        210        220        230        240        250        260        270
                   C DICGKRYKNR PGLSYHYAHT HLASEEGDEA QDQETRSPPN HRNENHRPQK GPDGTVIPNN YCDFCLGGSN

       280        290        300        310        320        330        340        350
MNKKSGRPEE LVSCADCGRS AHLGGEGRKE KEAAAAARTT EDLFGSTSES DTSTFHGFDE DDLEEPRSCR GRRSGRGSPT ADKKGSC
```

Figure S1 – Sequence composition of C-TERb (upper panel) and C-TERa (lower panel).

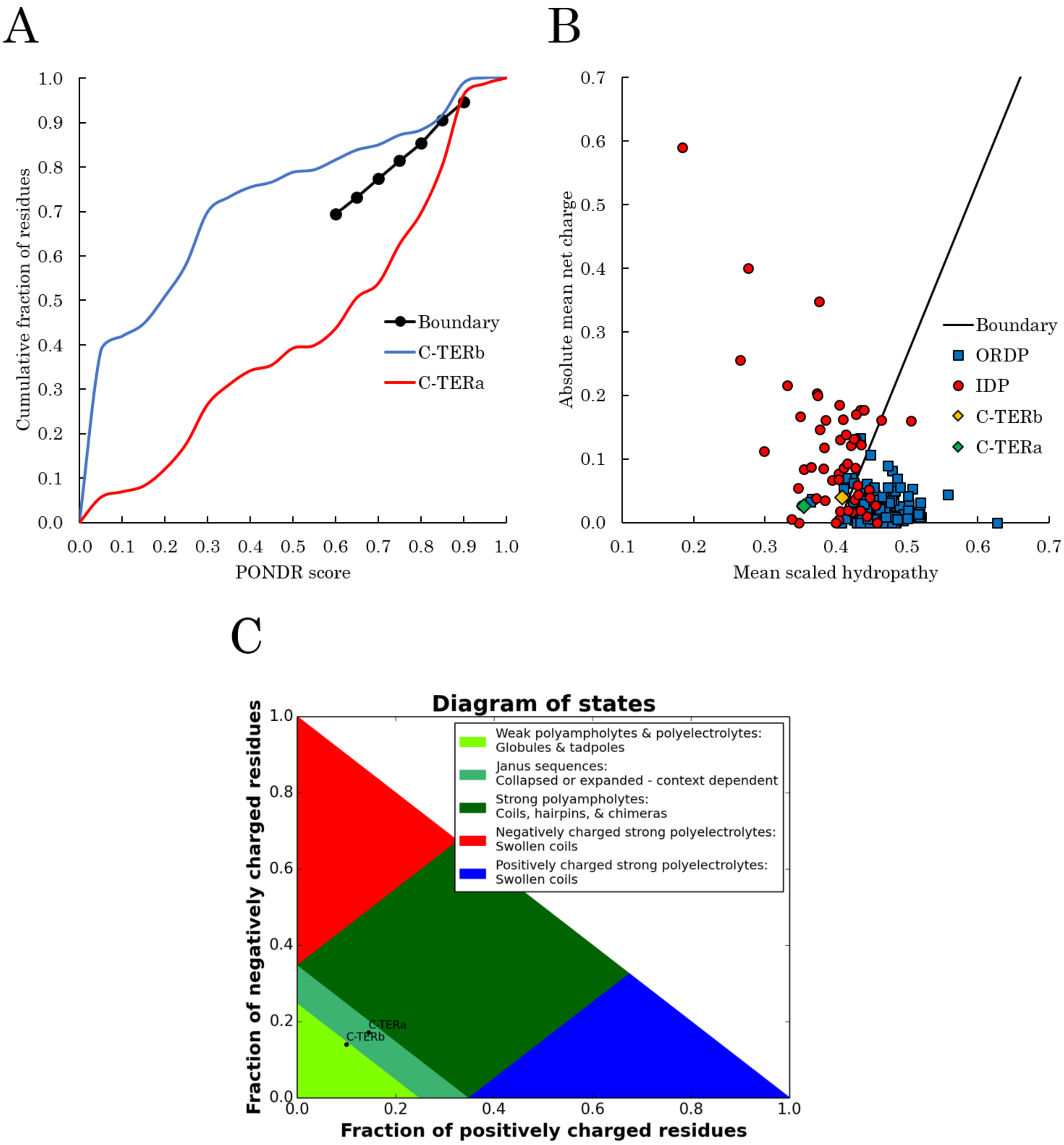

Figure S2 – Predicted disorder-associated properties of C-TERb and C-TERa. (A) CDF plot, (B) CH plot, and (C) Das-Pappu phase diagram.

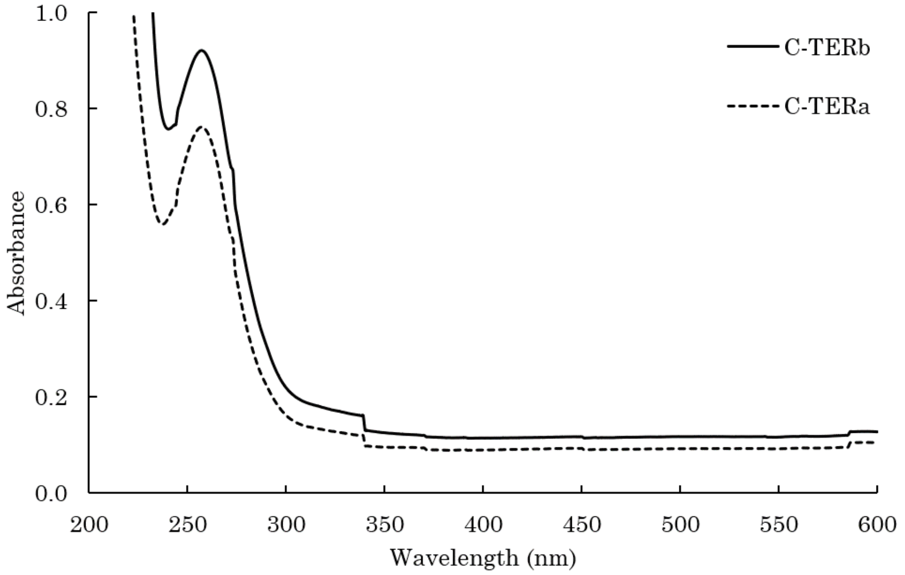

Figure S3 – UV-visible absorption spectra of C-TERb (solid line) and C-TERa (dashed line) in TBS at ~20 °C ($C_{C\text{-}TER}$ = 10 μM).

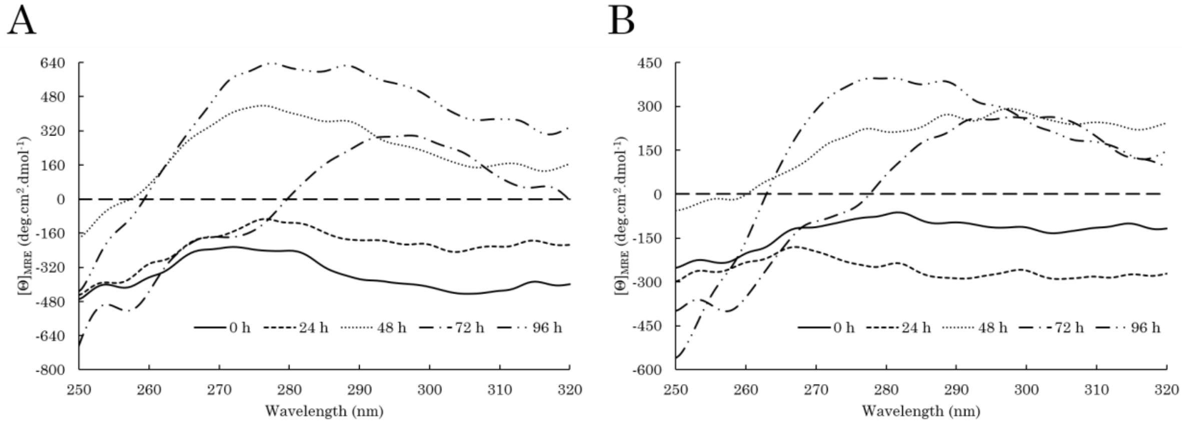

Figure S4 – Near-UV CD spectra of (A) C-TERb and (B) C-TERa ($C_{C\text{-}TER}$ = 10 μM). after 0 h (solid line), 24 h (dashed line), 48 h (dotted line), 72 h (dash-dotted line), and 96 h (dash-double-dotted line) of incubation in TBS at ~20 °C.

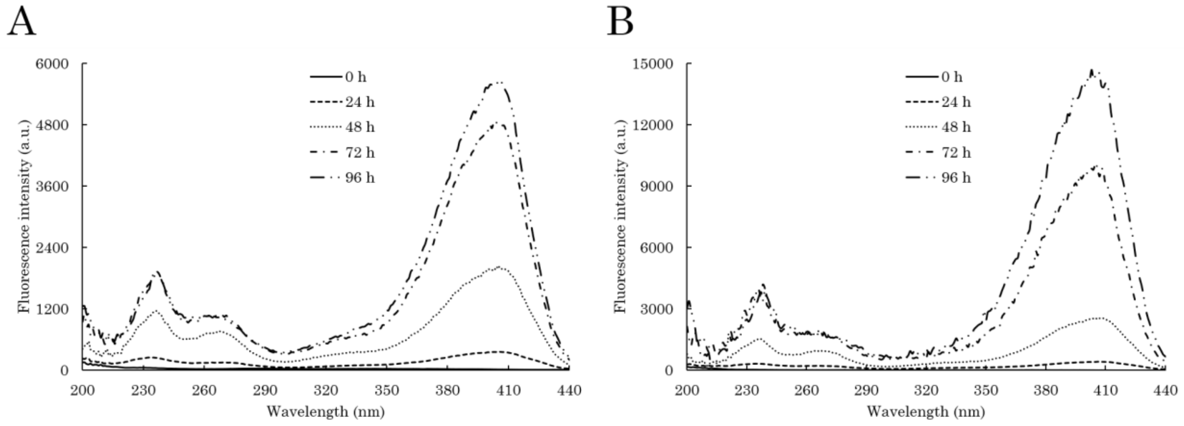

Figure S5 – dbAF excitation spectra (λ_{em} = 456 nm, sw = 10 nm) of (A) C-TERb and (B) C-TERa ($C_{C\text{-}TER}$ = 10 µM) after 0 h (solid line), 24 h (dashed line), 48 h (dotted line), 72 h (dash-dotted line), and 96 h (dash-double-dotted line) of incubation in TBS at ~20 °C.

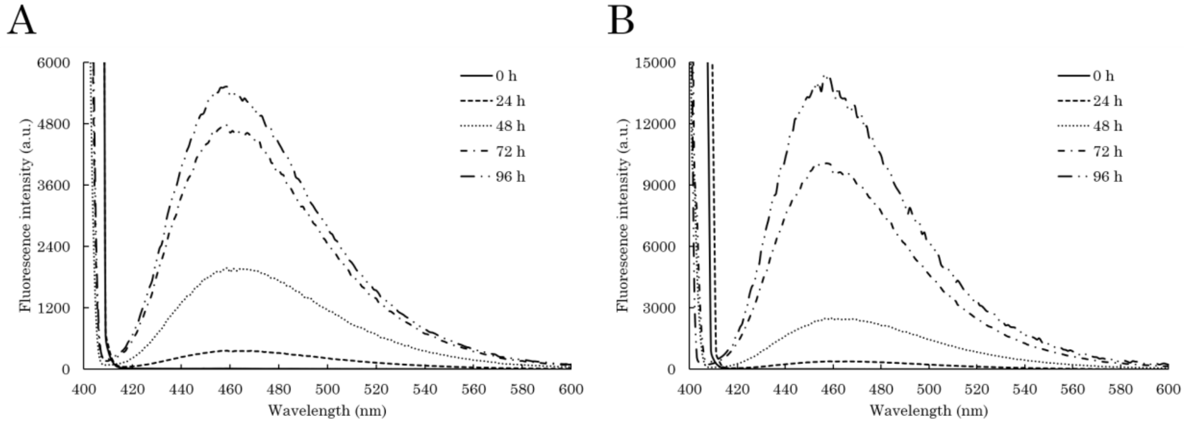

Figure S6 – dbAF spectra (λ_{ex} = 400 nm, sw = 10 nm) of (A) C-TERb and (B) C-TERa ($C_{C\text{-}TER}$ = 10 µM) after 0 h (solid line), 24 h (dashed line), 48 h (dotted line), 72 h (dash-dotted line), and 96 h (dash-double-dotted line) of incubation in TBS at ~20 °C.

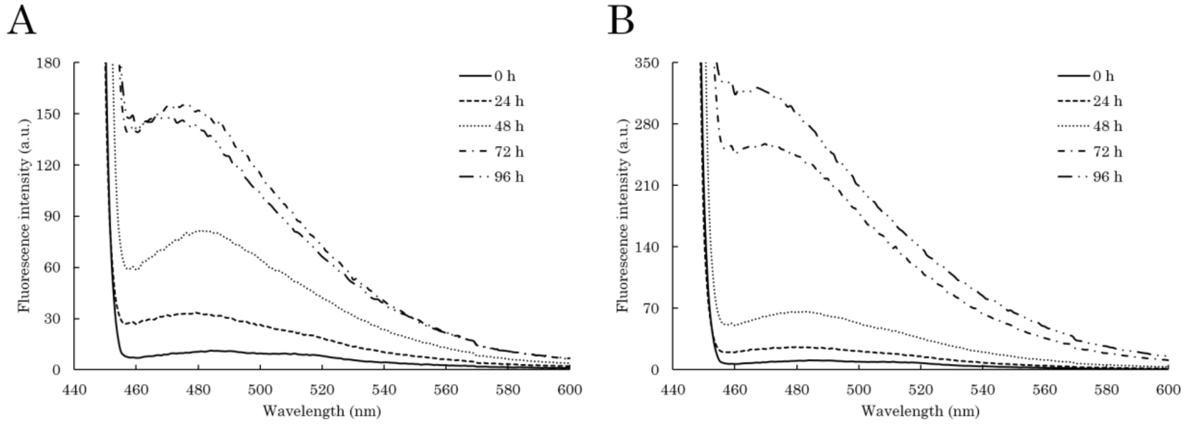

Figure S7 – ThT emission spectra (λ_{ex} = 440 nm, sw = 10 nm, C_{ThT} = 10 µM) of (A) C-TERb and (B) C-TERa ($C_{C\text{-}TER}$ = 10 µM) after 0 h (solid line), 24 h (dashed line), 48 h (dotted line), 72 h (dash-dotted line), and 96 h (dash-double-dotted line) of incubation in TBS at ~20 °C.

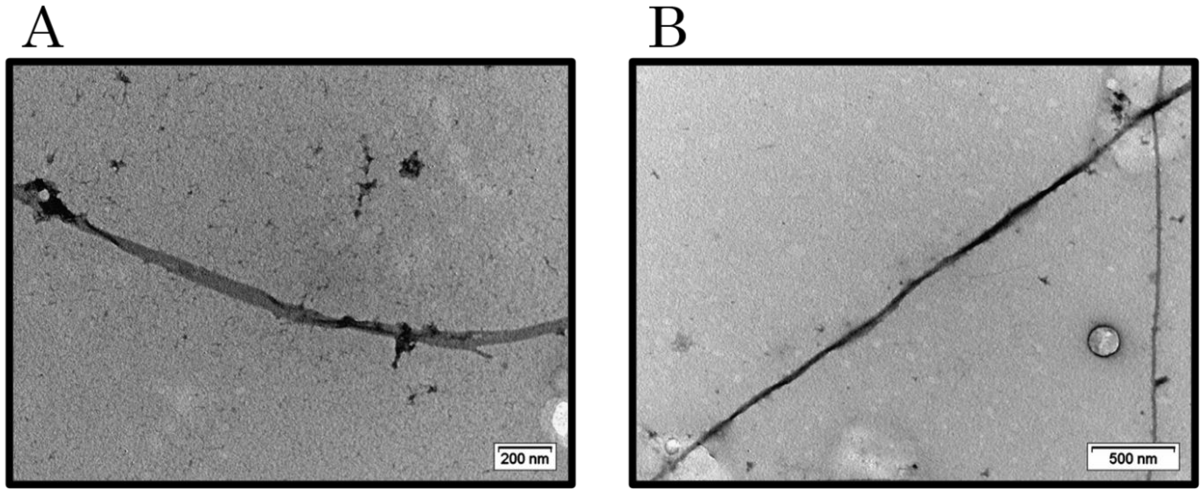

Figure S8 – Negatively stained TEM micrographs of (A) C-TERb and (B) C-TERa ($C_{C\text{-}TER}$ = 10 µM) thin twisted fibrils (TTFs) after 144 h (6 days) of incubation in TBS at ~20 °C. On each micrograph, the scale bar is indicated at the bottom right.

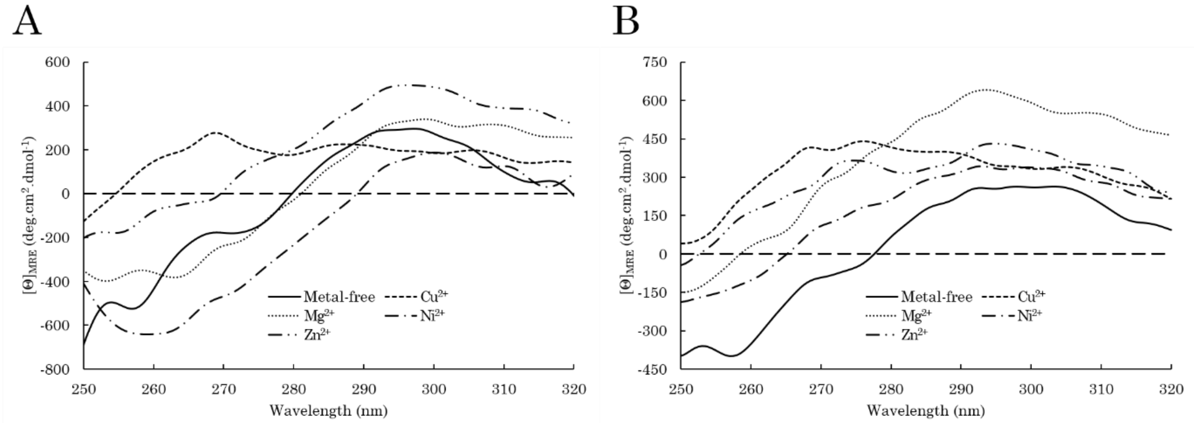

Figure S9 – Near-UV CD spectra of (A) C-TERb and (B) C-TERa ($C_{C\text{-}TER}$ = 5 µM) incubated in TBS at ~20 °C for 72 h without metal (solid line) and in the presence of Cu^{2+} (dashed line), Mg^{2+} (dotted line), Ni^{2+} (dash-dotted line), and Zn^{2+} (dash-double-dotted line) cations ($C_M{}^{2+}$ = 100 µM).

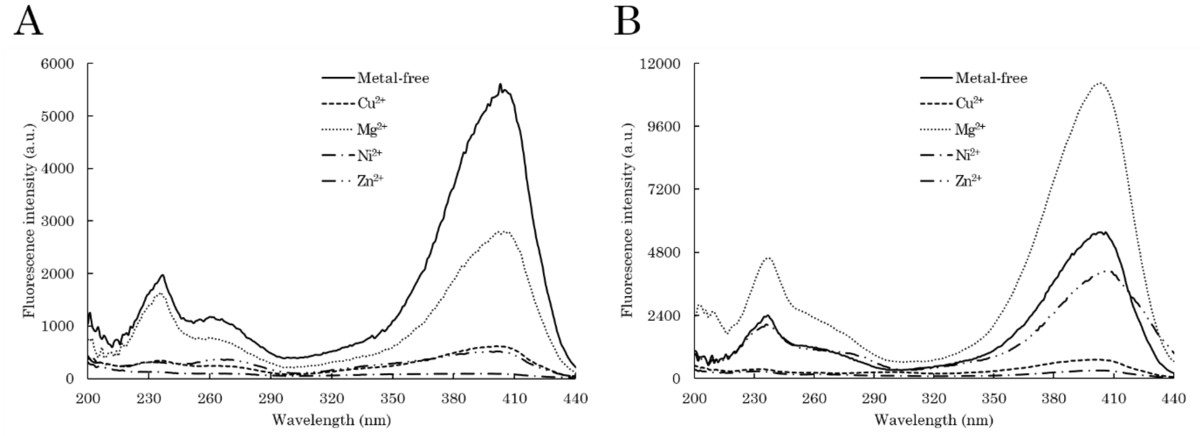

Figure S10 – dbAF excitation spectra (λ_{em} = 456 nm, sw = 10 nm) of (A) C-TERb and (B) C-TERa ($C_{C\text{-}TER}$ = 5 µM) incubated in TBS at ~20 °C for 72 h without metal (solid line) and in the presence of Cu^{2+} (dashed line), Mg^{2+} (dotted line), Ni^{2+} (dash-dotted line), and Zn^{2+} (dash-double-dotted line) cations ($C_M{}^{2+}$ = 100 µM).

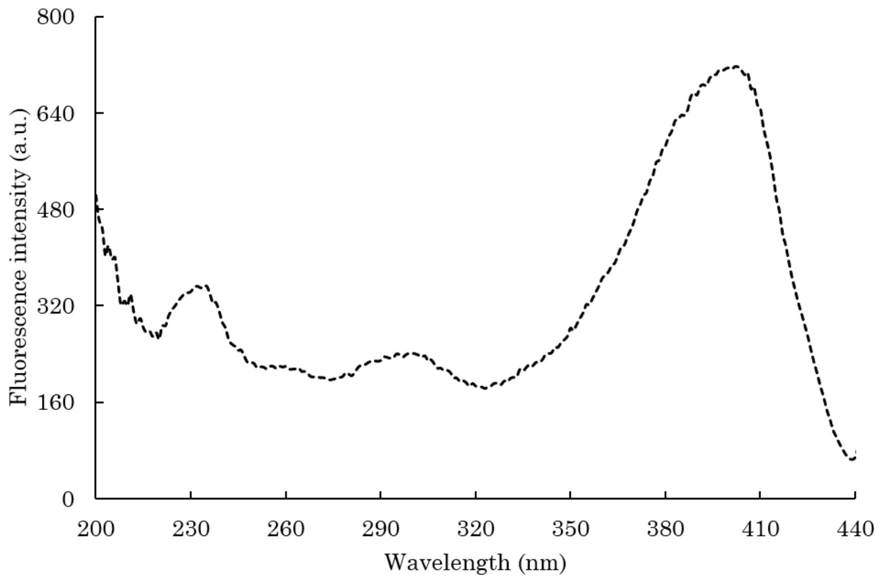

Figure S11 – dbAF excitation spectrum (λ_{em} = 456 nm, sw = 10 nm) of C-TERa ($C_{C\text{-}TERa}$ = 5 μM) incubated in TBS at ~20 °C for 72 h in the presence of Cu^{2+} ($C_{Cu}{}^{2+}$ = 100 μM).

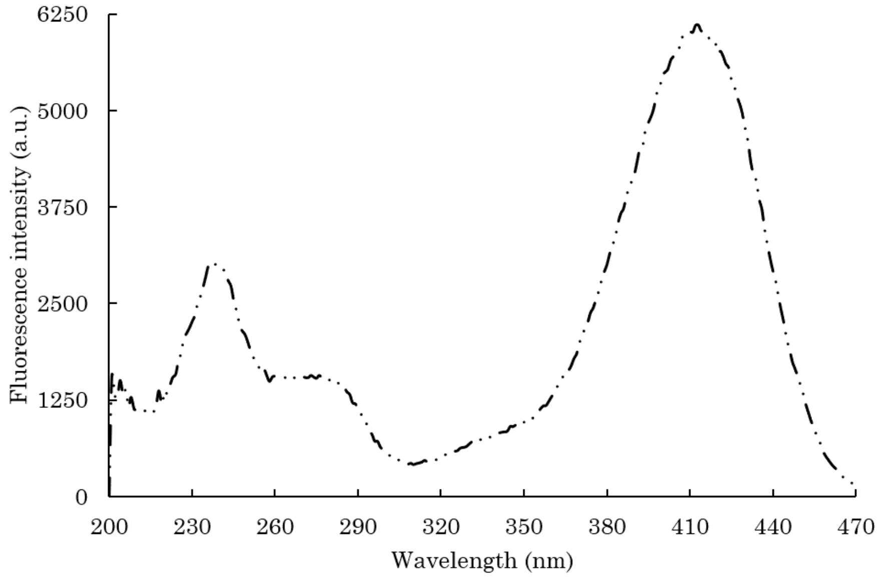

Figure S12 – dbAF excitation spectrum (λ_{em} = 490 nm, sw = 10 nm) of C-TERa ($C_{C\text{-}TERa}$ = 5 μM) incubated in TBS at ~20 °C for 72 h in the presence of Zn^{2+} ($C_{Zn}{}^{2+}$ = 100 μM).

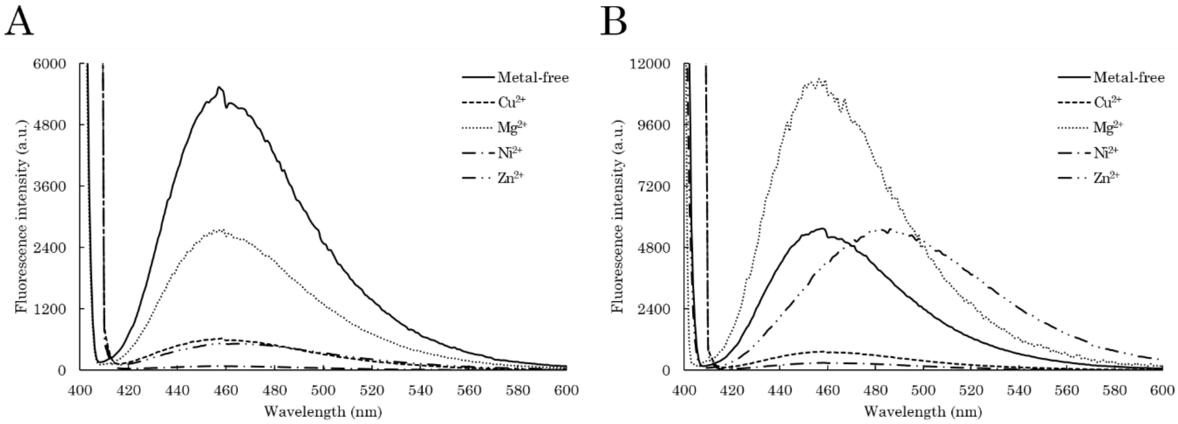

Figure S13 – dbAF spectra (λ_{ex} = 400 nm, sw = 10 nm) of (A) C-TERb and (B) C-TERa ($C_{C\text{-}TER}$ = 5 µM) incubated in TBS at ~20 °C for 72 h without metal (solid line) and in the presence of Cu^{2+} (dashed line), Mg^{2+} (dotted line), Ni^{2+} (dash-dotted line), and Zn^{2+} (dash-double-dotted line) cations ($C_M{}^{2+}$ = 100 µM).

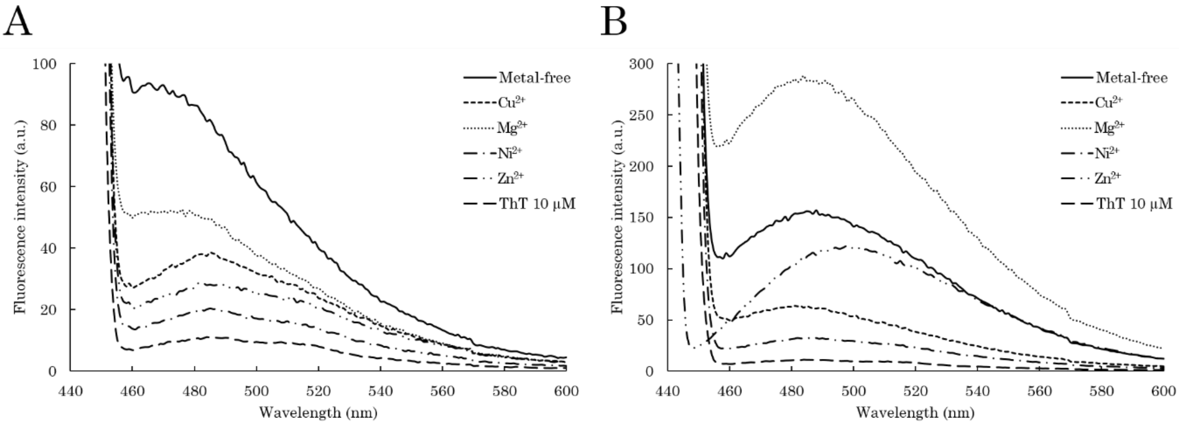

Figure S14 – ThT emission spectra (λ_{ex} = 440 nm, sw = 10 nm, C_{ThT} = 10 µM) of (A) C-TERb and (B) C-TERa ($C_{C\text{-}TER}$ = 5 µM) incubated in TBS at ~20 °C for 72 h without metal (solid line) and in the presence of Cu^{2+} (dashed line), Mg^{2+} (dotted line), Ni^{2+} (dash-dotted line), and Zn^{2+} (dash-double-dotted line) cations ($C_M{}^{2+}$ = 100 µM). The spectrum of ThT 10 µM alone in TBS is represented by the long-dashed line.

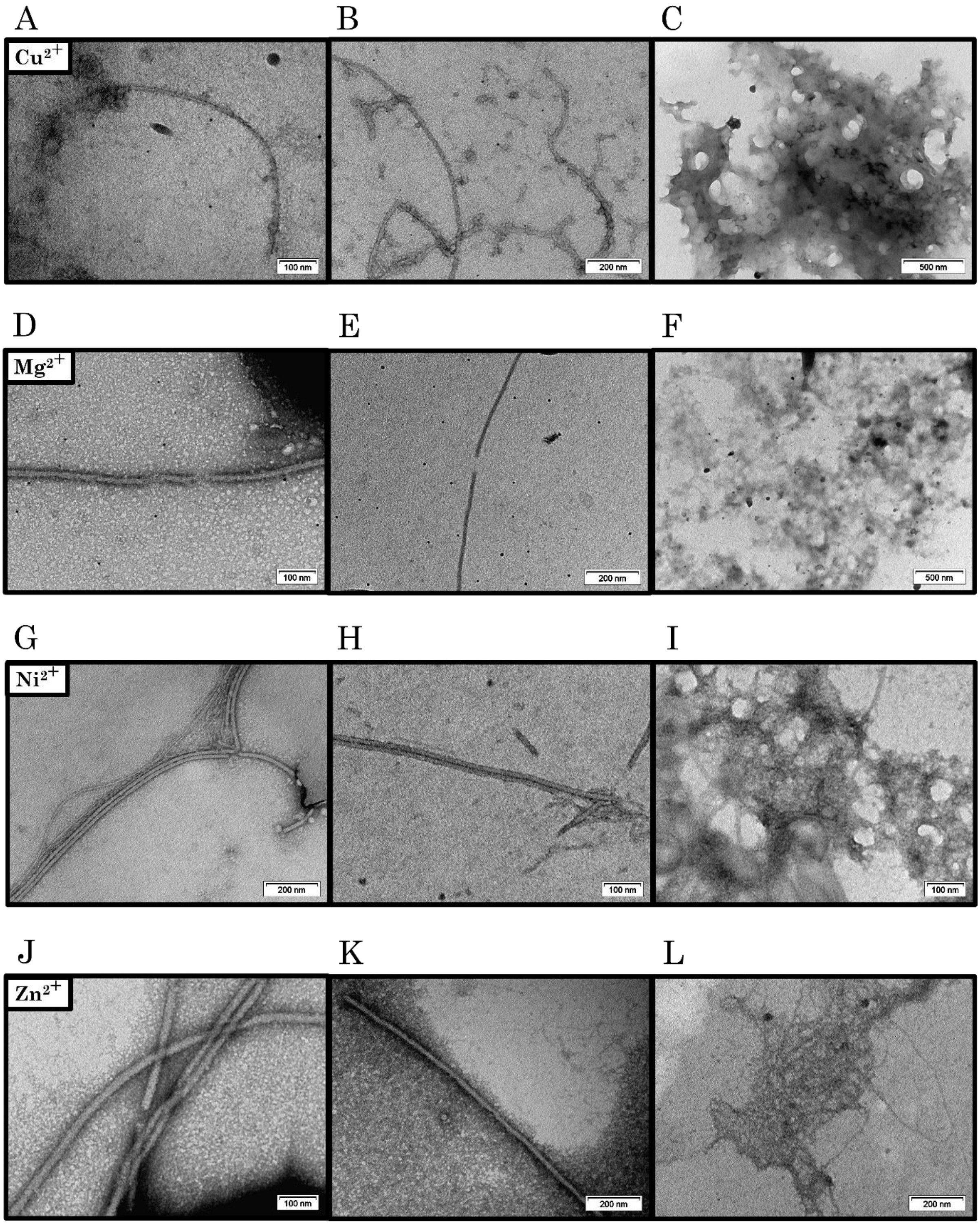

Figure S15 – Negatively stained TEM micrographs of C-TERb ($C_{C\text{-TERb}}$ = 5 µM) incubated for 144 h (6 days) in TBS at ~20 °C in the presence of (A, B, C) Cu^{2+}, (D, E, F) Mg^{2+}, (G, H, I) Ni^{2+}, and (J, K, L) Zn^{2+} cations (C_M^{2+} = 100 µM). Amorphous phases are observed in each condition (C, F, I, L). On each micrograph, the scale bar is indicated at the bottom right.

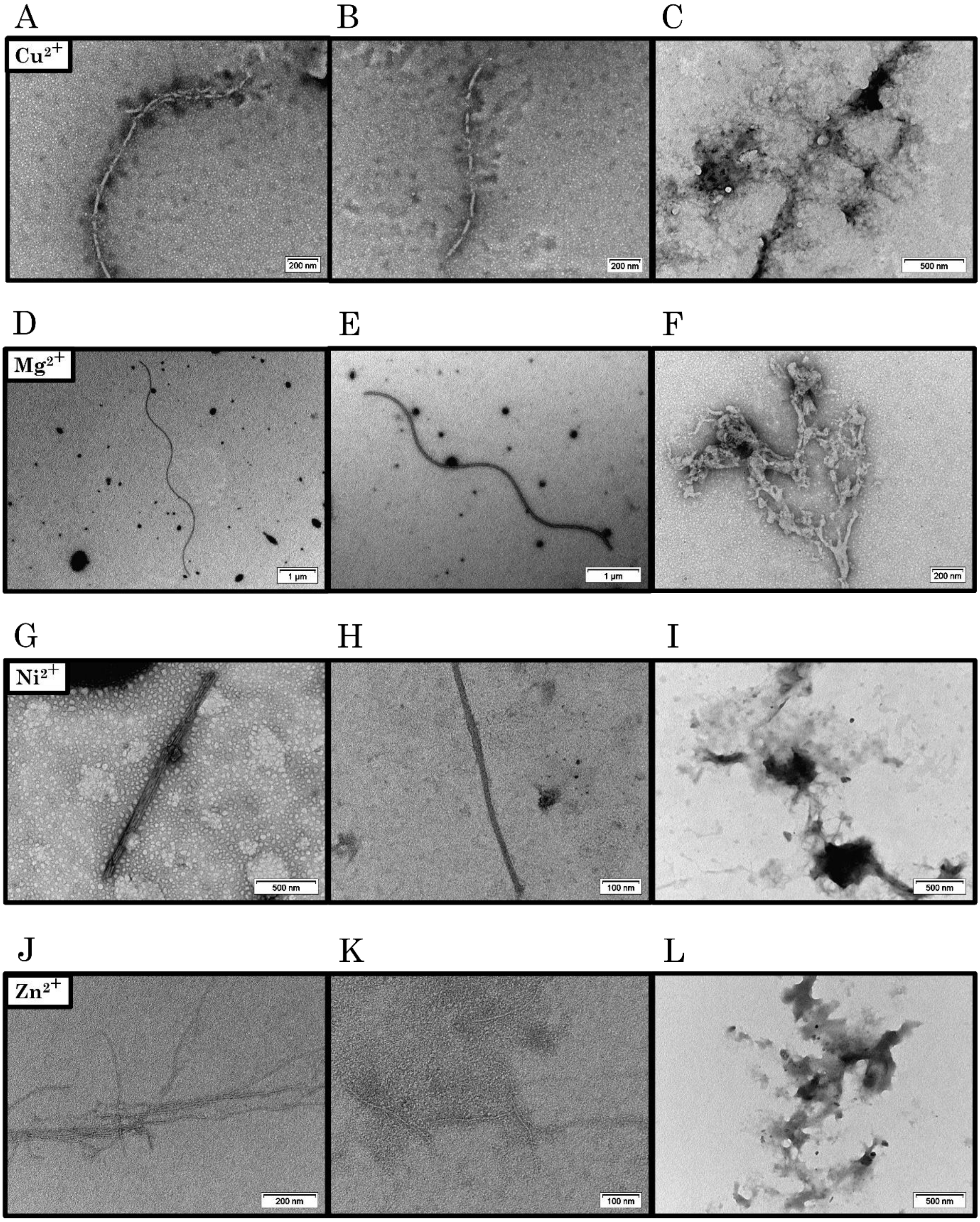

Figure S16 – Negatively stained TEM micrographs of C-TERa ($C_{C\text{-}TERa}$ = 5 µM) incubated for 144 h (6 days) in TBS at ~20 °C in the presence of (A, B, C) Cu^{2+}, (D, E, F) Mg^{2+}, (G, H, I) Ni^{2+}, and (J, K, L) Zn^{2+} cations ($C_{M}{}^{2+}$ = 100 µM). Amorphous phases are observed in each condition (C, F, I, L). On each micrograph, the scale bar is indicated at the bottom right.
